# Two distinct etiologies of gastric cardia adenocarcinoma: interactions among pH, *Helicobacter pylori*, and bile acids

**DOI:** 10.3389/fmicb.2015.00412

**Published:** 2015-05-11

**Authors:** Ken-ichi Mukaisho, Takahisa Nakayama, Tadashi Hagiwara, Takanori Hattori, Hiroyuki Sugihara

**Affiliations:** Division of Molecular Diagnostic Pathology, Department of Pathology, Shiga University of Medical Science, Otsu, Shiga, Japan

**Keywords:** *Helicobacter pylori*, cardia gastric adenocarcinoma, gastroesophageal reflux disease, bile acids, pH, atrophic gastritis

## Abstract

Gastric cancer can be classified as cardia and non-cardia subtypes according to the anatomic site. Although the gastric cancer incidence has decreased steadily in several countries over the past 50 years, the incidence of cardia cancers and esophageal adenocarcinoma (EAC) continue to increase. The etiological factors involved in the development of both cardia cancers and EACs are associated with high animal fat intake, which causes severe obesity. Central obesity plays roles in cardiac-type mucosa lengthening and partial hiatus hernia development. There are two distinct etiologies of cardia cancer subtypes: one associated with gastroesophageal reflux (GER), which predominantly occurs in patients without *Helicobacter pylori* (*H. pylori*) infection and resembles EAC, and the other associated with *H. pylori* atrophic gastritis, which resembles non-cardia cancer. The former can be developed in the environment of high volume duodenal content reflux, including bile acids and a higher acid production in *H. pylori*–negative patients. *N*-nitroso compounds, which are generated from the refluxate that includes a large volume of bile acids and are stabilized in the stomach (which has high levels of gastric acid), play a pivotal role in this carcinogenesis. The latter can be associated with the changing colonization of *H. pylori* from the distal to the proximal stomach with atrophic gastritis because a high concentration of soluble bile acids in an environment of low acid production is likely to act as a bactericide or chemorepellent for *H. pylori* in the distal stomach. The manuscript introduces new insights in causative factors of adenocarcinoma of the cardia about the role of bile acids in gastro-esophageal refluxate based upon robust evidences supporting interactions among pH, *H. pylori*, and bile acids.

## Introduction

Gastric cancer is the sixth most common type of cancer and the fourth most common cause of cancer-related deaths worldwide (Ferlay et al., 2013). Gastric cancer can be classified according to the anatomic site as cardia (the upper part of the stomach adjoining the esophagus) and non-cardia (the mid and distal stomach) subtypes. Although the incidence of gastric cancer has steadily declined in several countries over the past 50 years, the overall decline is opposite to the subsite-specific increase in cardia cancers (Chow et al., 1998b; Carr et al., 2013). The increase in incidence is approximately seven fold, a more substantial increase than that in several other malignancies (Carr et al., 2013). Potential differences in the presentation and outcome of patients with gastric cardia or non-cardia adenocarcinoma may exist (Shi et al., 2011). Although it has been reported that cardia cancers may be related to gastroesophageal reflux (GER) similar to esophageal adenocarcinoma (EAC), the etiology of adenocarcinoma of the cardia remains unclear and controversial (Ye et al., 2001).

It is widely accepted that chronic mucosal inflammation causes the majority of gastric cancers due to *Helicobacter pylori* (*H. pylori*) infection (International Agency for Research on Cancer, 1994). However, the roles of *H. pylori* infection in the development of cardia cancer are contradictory (Chow et al., 1998a; Eslick et al., 1999; Ekstrom et al., 2001; Helicobacter and Cancer Collaborative Group, 2001; Limburg et al., 2001; Kamangar et al., 2006, 2007). Several studies indicate two distinct etiologies of cardia cancer: one that is more commonly associated with GER in *H. pylori*–negative than in *H. pylori*–positive patients and resembles EAC; and the other that is associated with *H. pylori* atrophic gastritis and resembles non-cardia cancer (McColl, 2006; Hansen et al., 2007; Derakhshan et al., 2008). In this manuscript, we try to explain the potential mechanisms involved in the development of gastric cardia adenocarcinoma with two distinct etiologies by elucidating the interaction among pH, *H. pylori*, and bile acids.

## Cardia Adenocarcinoma Associated with GER Resembling EAC

Here we attempt to explain the possible mechanism involved in the development of cardia adenocarcinoma associated with GER by considering the causative factors of EAC.

### Gastroesophageal Reflux Disease and Barrett Esophagus: Major Risk Factors for EAC

Most cases of esophageal cancer arise as squamous cell carcinoma or adenocarcinoma. Forty years ago, approximately 75% of diagnosed esophageal cancer cases, even those in the United States (U.S.), were esophageal squamous cell carcinoma and the remaining 25% were EAC. Among Caucasian men, the incidence of EAC has increased since the mid-1970s, and EAC has become the leading type of esophageal cancer, representing 80% of cases (Devesa et al., 1998; Enzinger and Mayer, 2003; Absi et al., 2013).

Gastroesophageal reflux disease (GERD) and Barrett esophagus are major risk factors for EAC, the risk for which increases with the frequency and duration of reflux symptoms (Lagergren et al., 1999; Wu et al., 2003; Anandasabapathy et al., 2007). Frequent reflux of the gastric juices, which contain acid, pepsin, and bile, is thought to induce Barrett esophagus, a condition of columnar metaplasia of the esophageal squamous epithelium damaged by GER (Souza et al., 2002; Theisen et al., 2005; Spechler and Souza, 2014). U.S. gastroenterology societies require esophageal biopsies to show intestinal metaplasia with goblet cells for the definitive diagnosis of Barrett esophagus (Wang and Sampliner, 2008; Spechler et al., 2011; ASGE Standards of Practice Committee et al., 2012). It has been widely accepted that an intestinal-type mucosa is the precursor of EAC (Ruol et al., 2000). However, it has been recently accepted that a cardiac-type mucosa without the intestinal goblet cells background of EAC is also occasionally encountered (Takubo et al., 2005, 2009; Kushima et al., 2013).

### Roles of Central Obesity in Cardiac-type Mucosa Lengthening and Partial Hiatus Hernia Development

Abdominal obesity promotes GERD by elevating intra-abdominal pressure, which produces a hiatus hernia (El-Serag, 2008). Studies comparing computed tomography–measured abdominal fat composition showed that a large amount of visceral abdominal fat relative to subcutaneous fat is associated with a significant increase in the risk of Barrett esophagus (El-Serag et al., 2014). A recent study of 24 healthy *H. pylori*–negative volunteers with a small waist circumference and 27 with a large waist circumference reported that central obesity is associated with the intrasphincteric extension of gastric acid, allowing it to move proximally within the intrasphincteric reflux, and cardiac-type mucosal lengthening, which predisposes subjects to cardia adenocarcinoma (Robertson et al., 2013). Furthermore, according to a recent study of healthy volunteers without a history of GERD, central obesity and the use of a waist belt can produce a partial hiatus hernia and contribute to excess acid exposure of the distal esophageal mucosa, leading to metaplastic columnar changes (Lee et al., 2014).

### Role of High Animal Fat Intake in EAC Development

A high fat intake, which can be a cause of central obesity, plays a role in an increased risk of GERD symptoms and erosive esophagitis, which are risk factors for EAC (El-Serag et al., 2005). The mechanism underlying this effect remains unclear; however, it may be proven by the increasing rates of GERD in the U.S. because the fat content of its food supply has increased by 38% between 1909 and 1988 (Raper et al., 1992). To examine the roles of high animal fat intake in the etiology of EAC, we fed a high animal fat diet to rats with reflux of the duodenal contents (Chen et al., 2007). First, Wistar rats in the control group were fed a low soybean oil diet and the other rats in the high-fat group were fed a high cow fat diet. The resulting morphological changes between these groups were then evaluated up to 30 weeks after surgery. The rats with reflux of the duodenal contents in the high-fat group showed a significantly higher incidence of Barrett esophagus and Barrett dysplasia than those in the control group. In addition, the incidence of EAC in the high-fat group was also slightly higher than that in the control group (Chen et al., 2007). These findings suggest that a high animal fat intake plays a pivotal role in EAC. In the clinical setting, it has been reported that high-fat dairy is associated with an increased risk of EAC (Navarro Silvera et al., 2008).

### High Dietary Animal Fat Changes the Bile Acid Composition

We compared the total level and composition of bile acids in the bile juice collected from the common bile ducts of rats that had not undergone surgery (Chen et al., 2007). The bile juice was aspirated after the animals had been fed different diets for 1 month, and the bile acid concentrations were measured. High dietary animal fat changed the bile acid composition and increased the concentration of taurine conjugates in the bile juice (Chen et al., 2007). The chemical characteristics of bile in humans are governed by the *pKa* of the individual bile acids. Free bile acids have a *pKa* of approximately 7 and comprise approximately 2% of the bile. Glycine-conjugated bile acids have a *pKa* of 4.3–5.2 and comprise >60% of the bile, while taurine-conjugated bile acids have a *pKa* of 1.8–1.9 and comprise 20% of the bile, resulting in a ratio of glycine to taurine conjugates of approximately 3:1 (Nair and Kritchenvski, 1971; Stamp, 2002). Because only taurine conjugates are soluble among various bile acids in humans with intact gastric acid production and can affect the epithelium, an increase in taurine-conjugated bile acids in the refluxate under acidic conditions may play a role in Barrett carcinogenesis and cancer progression.

In a study examining bile acid, the esophageal samples taken from 10 asymptomatic subjects and 30 patients with GERD symptoms were analyzed using modified high-performance liquid chromatography (Nehra et al., 1999). There was a significantly greater proportion of secondary bile acids, deoxycholic acids, and taurodeoxycholic acids in patients with erosive esophagitis and Barrett esophagus/stricture. Taurocholic acid was also significantly increased in the Barrett esophagus/stricture group compared with the minimal injury group (Nehra et al., 1999). The study concluded that mixed reflux is more harmful than acid reflux alone and that possible toxic synergism exists between the taurine conjugates and the acid (Nehra et al., 1999). These findings are consistent with those of our animal experiments mentioned above.

### Endogenous DNA Adducts and *N*-nitroso Bile acids

To explore the causative factors of EAC, thiazolidine-4-carboxylic acid (thioproline) was administered as a nitrite scavenger to rats considered the gastroduodenal contents reflux model (Kumagai et al., 2004). Postoperatively, we divided the reflux animals into two diet groups. Animals in the control group were given a normal diet, while the thioproline group was given food containing 0.5% thioproline. All esophageal sections in both groups were histologically evaluated. EAC developed in 38.9% of the control group, whereas no EAC was detected in the thioproline group (Fisher exact test, *P* < 0.05). We then proposed that nitroso compounds derived from duodenal contents reflux play a crucial role in the development of EAC (Kumagai et al., 2004). We also performed a similar experiment using the rat duodenal contents reflux model for gastric carcinogenesis. This study also suggested a connection between nitroso compounds and gastric carcinogenesis (Suo et al., 2006). Furthermore, Terasaki et al. (2008) detected the endogenous DNA adducts produced from *N*-nitroso bile acid conjugates, such as *N*-nitrosoglycocholic acid and *N*-nitrosotaurocholic acid, in the glandular stomach of the duodenal contents reflux rats. These observations confirmed that *N*-nitrosotaurocholic acid and *N*-nitrosoglycocholic acid are formed by the nitrosation of glycocholic acid and taurocholic acid, respectively. These studies suggest that nitrosated bile acid conjugates, which have mutagenicity, could contribute to the development of both EAC and gastric carcinogenesis.

### Highly Acidic Condition Stabilizes Mutagenic *N*-nitroso Bile Acids

The pathogenesis of GERD is multifactorial since it involves transient lower esophageal sphincter relaxation as well as other lower esophageal sphincter pressure abnormalities (Castell et al., 2004). GERD is a complex problem caused by many factors that are exacerbated when the patient is in the supine position (Castell et al., 2004). Gastric contents are easily pooled, and *N*-nitroso compounds could be generated at night, in the gastric juice with the refluxate including bile acids in the fornix located on the posterior side of the body. Moreover, both the esophagus and the proximal cardia portion are easily exposed to the *N*-nitroso compounds by transient lower esophageal sphincter relaxation as well as other lower esophageal sphincter pressure abnormalities in the supine position (Macke et al., 2011). We reported that one of these nitroso compounds, *N*-nitroso glycocholic, rapidly decomposed under alkaline conditions (pH 9) [t_(1/2)_ = 0.96 h] but remained quite stable under acidic (pH 2) [t_(1/2)_ = 12.8 h] and neutral (pH 7) [t_(1/2)_ = 7.8 h] conditions (Araki et al., 2008). These findings suggest that a highly acidic condition also contributes to carcinogenesis as a stabilizer of *N*-nitroso bile acids.

### *H. pylori* Protects Against GERD

There are inverse associations between the presence of *H. pylori* (particularly cagA^+^ strains) and disorders such as GERD, Barrett esophagus, and EAC (Vicari et al., 1998; Peek et al., 1999; Vaezi et al., 2000; Ye et al., 2004; Islami and Kamangar, 2008), suggesting a protective role of *H. pylori*. One potential mechanism for this effect could be that *H. pylori* colonization diminishes gastric acidity; therefore, during reflux episodes, the highly acidic refluxate in *H. pylori*–negative patients may be more damaging to the esophageal epithelium than the low acidic refluxate from the stomach with *H. pylori*–associated atrophic gastritis.

## Cardia Adenocarcinoma Associated with *H. pylori* Atrophic Gastritis Resembling Non-cardia Cancer

Here we attempt to explain the possible mechanism of the development of cardia adenocarcinoma resembling non-cardia cancer by considering the interactions among pH, *H. pylori*, and bile acids.

### Relationship between *H. pylori* and Non-cardia Gastric Carcinogenesis

*H. pylori* generally colonizes to the pyloric antrum and *H. pylori* was designated a class I carcinogen by the World Health Organization (International Agency for Research on Cancer, 1994). Thus, the best-established risk factor for non-cardia gastric cancer is *H. pylori* infection (Kamangar et al., 2006; Brenner et al., 2009). However, *H. pylori* infection is required for gastric cancer to develop but not for gastric carcinogenesis. There are several animal models of *H. pylori*–induced carcinogenesis, but unless the bacteria are combined with a chemical carcinogen or hypergastrinemia, none of these models reliably produce a malignancy similar to that observed in humans (Hagiwara et al., 2011; Hayakawa et al., 2013; Tsukamoto et al., 2013; Yu et al., 2014; Graham, 2015). *H. pylori* has been shown to promote the production of inflammatory mediators such as interleukin-1β and tumor necrosis factor-α, potent suppressors of stomach juice secretion. The increase in stomach pH may lead to the spread of *H. pylori* from the antrum to the corpus, resulting in enhanced inflammation in the mucosa of the corpus followed by parietal cell destruction and irreversible hypochlorhydria (Takashima et al., 2001; Peek and Blaser, 2002).

### Cardia Adenocarcinoma Associated with *H. pylori* Atrophic Gastritis

Although most studies of Western populations found no association or an inverse association, few studies of Asian populations have found a positive association between *H. pylori* seropositivity and cardia cancer (Helicobacter and Cancer Collaborative Group, 2001; Dawsey et al., 2002). Recently, two studies reported on the origin of gastroesophageal junction adenocarcinoma or esophagogastric junction, which are similar meaning to cardia adenocarcinoma. Horii et al. reported that the two distinct types of cancer of different origin might be mixed in cardia adenocarcinoma: Barrett esophageal cancer might be associated with high gastric acid secretion and reflux of gastric acid into the esophagus, and cancer resembling distal gastric cancer might be associated with gastric atrophy and low gastric acid secretion (Horii et al., 2011). Yamada et al. reported that approximately half of patients with cardia adenocarcinoma harbor histological gastritis (Yamada et al., 2014). These findings suggest that a part of cardia adenocarcinoma can be associated with *H. pylori* atrophic gastritis resembling non-cardia cancer in especially Asian countries.

### Interactions Among Bile Acids, pH, and *H. pylori*

#### Mechanism of Duodenal Ulcer Treatment by Antisecretory Therapy

The combination of a high duodenal acid load and *H. pylori* infection is possibly a critical event in the pathogenesis of *H. pylori*–related duodenal ulcer disease (Graham and Osato, 2000). Graham et al. suggested that, because glycine-conjugated bile acids are precipitated at an acidic pH, *H. pylori* can survive in gastric metaplasia and any event that leads to an increase in the duodenal acid load predisposes patients with *H. pylori* infection to duodenal ulcer diseases (Graham et al., 1996). They and other authors also reported that any condition that reduces the duodenal acid load (e.g., antisecretory therapy) allows the bile acids to remain in the solution, inhibits the growth of *H. pylori*, and promotes ulcer healing (Han et al., 1996; Graham and Osato, 2000).

#### Long-term use of Proton Pump Inhibitors Exacerbates Corpus Atrophic Gastritis in *H. pylori*–positive Patients

*H. pylori* predominantly colonizes the gastric antrum in subjects in whom acid production is intact. In contrast, in subjects in whom acid production is decreased by whatever mechanism, including the use of *proton pump inhibitors* (PPIs), *H. pylori* colonizes the body of the stomach (Kuipers, 2006). Although there is no proof that the PPI use increases the risk of gastric cancer at present, PPI therapy affects the pattern and severity of *H. pylori* gastritis and accelerates the process of corpus gland loss (Kuipers, 2006). We recently proposed the mechanism of how the long-term use of PPIs exacerbates corpus atrophic gastritis in *H. pylori*–positive patients with GERD that includes interactions among bile acids, pH, and *H. pylori* (Mukaisho et al., 2014; Hagiwara et al., 2015). The original idea about the mechanism of duodenal ulcer treatment by antisecretory therapy was proposed in the papers mentioned above.

Duodenogastric reflux including bile reportedly occurs even in healthy individuals (Keane et al., 1981; Sonnenberg et al., 1982; Thompson, 1982; Heading, 1983; Muller-Lissner et al., 1983), and bile reflux is increased in patients with GERD (Kauer et al., 1997). Bile acids are significant chemorepellents for the bacillus *H. pylori* (Worku et al., 2004). PPIs administration inhibits gastric acid production and changes the concentration of soluble bile acids in the stomach. Because taurine conjugates have a *pKa* of 1.8–1.9 and glycine conjugates have a *pKa* of 4.3–5.2, only taurine conjugates are soluble in an acidic environment. However, glycine conjugates as well as taurine conjugates become soluble in the presence of PPIs administration. Because the concentration of soluble bile acids in patients with GERD on PPI therapy is considerably higher than that in normal subjects with intact acid production, especially in the distal stomach with duodenogastric reflux including bile, the colonization of *H. pylori* changes the pattern from antral-predominant to corpus-predominant (Mukaisho et al., 2014).

#### Mechanism of Changing Colonization of *H. pylori* in Atrophic Gastritis

The similar phenomena may occur in the stomach with *H. pylori*–associated atrophic gastritis because this condition induces an environment of low acid production in the stomach. The high concentration of soluble bile acids in the environment of low acid production, especially in the distal stomach, is likely to act as a bactericide or chemorepellent for *H. pylori*. In contrast, the concentration of soluble bile acids in the proximal stomach is less than that of distal stomach. *H. pylori* may then colonize within the proximal rather than distal stomach.

Furthermore, it has been reported that the development of atrophic gastritis late in life diminishes or eliminates *H. pylori* colonization (Karnes et al., 1991). Potential reasons for the specific association of *H. pylori* with a normal gastric mucosal epithelium include a low pH requirement for metabolic processes; dependence on specific nutrients, mucins, or cell-surface components that are specific features of the gastric epithelium; or the inability to compete in environments in which other microbes are more abundant. These findings also might explain the phenomenon of the changing colonization of *H. pylori* from the distal to the proximal stomach.

## Summary

Based on anatomic site, gastric cancer can be classified as cardia or non-cardia subtypes. There are two distinct etiologies of cardia cancer: one associated with GER and resembles EAC, and the other associated with *H. pylori* atrophic gastritis and resembles non-cardia cancer. The former can develop in the environment of high volume duodenal contents reflux, which includes bile acids and a higher acid production in *H. pylori*–negative than in *H. pylori*–positive patients. The latter can develop because of the changing colonization of *H. pylori* from the distal to the proximal stomach with atrophic gastritis in patients with *H. pylori* infection. Although the reason for the striking increase in the proportion of cases of cardia cancer as well as EAC remains unknown, it may at least partially be explained by increasing rates of GERD, which induces duodenal contents reflux into the stomach.

### Conflict of Interest Statement

The authors declare that the research was conducted in the absence of any commercial or financial relationships that could be construed as a potential conflict of interest.
